# The relationship between sleeptime and depression among middle-aged and elderly Chinese participant during COVID-19 epidemic and non-epidemic phases

**DOI:** 10.3389/fpsyt.2024.1361184

**Published:** 2024-05-10

**Authors:** Chaonan Du, Cong Wang, Zhiwei Liu, Nan Bai, Junhao Zhu, Alleyar Ali, Yuanming Geng, Xinrui Zeng, Yu Yang, Zhenxing Li, Chiyuan Ma

**Affiliations:** ^1^ Nanjing Jinling Hospital, Affiliated Hospital of Medical School, Nanjing University, Nanjing, China; ^2^ Nanjing Jinling Hospital, Nanjing University of Chinese Medicine, Nanjing, Jiangsu, China; ^3^ Department of Neurosurgery, The Affiliated Jinling Hospital of Nanjing Medical University, Nanjing, China; ^4^ School of Medicine, Southeast University, Nanjing, Jiangsu, China

**Keywords:** sleeptime, age, elderly, depression, epidemic

## Abstract

**Background:**

The global impact of the COVID-19 pandemic had significantly altered the daily routines of people worldwide. This study aimed to compare how sleeptime and depression among Chinese residents had differed between periods during and outside the epidemic. Furthermore, it delved into the interactive effect of age in this relationship.

**Method:**

Utilizing data from the China Health and Retirement Longitudinal Study (CHARLS) study in 2015 and the recently released data from 2020, which covered the pandemic period. Depression was assessed using Center for Epidemiologic Studies Depression Scale (CESD-10), considering a score of 10 or higher as indicative of depression. Participants were categorized based on age, specifically those aged 60 years and older. multivariate logistic regression and interaction analyses were employed to assess the interplay of age, supported by subgroup and sensitivity analyses to reinforce our findings.

**Results:**

The 2020 database comprised 19,331 participants, while the 2015 database had 10,507 participants. Our findings demonstrated a significant correlation between sleeptime and depression in both unadjusted models and models adjusted for all variables in both datasets (p<0.001). Upon stratifying by age and adjusting for relevant factors, we identified an interaction effect among age, sleeptime, and depression (p=0.004 for the interaction in the 2020 database, compared to 0.004 in 2015). The restricted cubic spline analysis in both datasets showcased a nonlinear relationship between sleeptime and depression.

**Conclusions:**

During both epidemic and non-epidemic periods in China, there existed a correlation between sleep duration and depression, which interacts with age.

## 1 Introduction

Depression stands as a significant global public health challenge, leaving a profound impact on populations worldwide. Statistics reveal that roughly 350 million individuals globally grapple with depression, underscoring its grave threat to overall societal well-being (1). Meanwhile, sleep, a pivotal factor in maintaining physical health, correlates closely with chronic conditions like cardiovascular and metabolic diseases (2, 3). In China, among elderly community members, insomnia rates range from 24% to 59% (4, 5). Many in this group turn to sleep medications for extended periods, yet these drugs often fail to meaningfully enhance sleep quality for the majority of patients (6). Prolonged insomnia can exacerbate mental health issues such as depression and anxiety, amplifying the burden on those affected (7). The intimate link between declining sleep quality and mental health concerns underscores the pressing need for an in-depth exploration of this matter. A deeper grasp of the interplay between insomnia and depression, along with comprehending insomnia’s long-term health impacts, can pave the way for more effective intervention strategies. This, in turn, could enhance sleep quality and alleviate the weight of mental health challenges.

The past bout of COVID-19 has sparked seismic shifts in people’s lifestyles, reshaping work routines, social engagements, and daily activities, potentially deeply impacting sleep and mental well-being. Studies reveal that numerous individuals experienced significant disruptions in their sleep patterns amid the pandemic. Factors like social isolation, anxiety, and information overload have led to erratic sleep schedules and diminished quality. This shift in sleep habits closely links to heightened risks of depression. Altena et al (8) observed that amidst social isolation, individuals grapple with sleep issues, likely stemming from lifestyle shifts and heightened emotional strain. Moreover, Cellini et al (9) noted changes in sleep behaviors among Italian residents during the COVID-19 lockdown, indicating that increased social isolation and heightened digital media usage might correlate with declining sleep quality. The COVID-19 outbreak has impacted the ongoing treatment of depression patients. Constraints on accessing medical facilities, disruptions in psychological counseling, and interruptions in drug supplies may hinder patients from timely care, further worsening depression symptoms and severity.

Thus, exploring the correlation between sleeptime and depression during COVID-19 and non COVID-19 underscores the critical tie between sleep quality and mental well-being. Interventions addressing sleep challenges could potentially mitigate depression risks and enhance individual mental health.

## 2 Methods

### 2.1 Data sources and variables collection

We utilized data from the China Longitudinal Study on Health and Retirement (CHARLS), encompassing content published in 2015 and 2020. The CHARLS participant encompassed over 100 randomly selected counties and districts nationwide, with respondents representing both urban and rural areas, making it highly representative of China’s middle-aged and elderly population. The CHARLS dataset for 2020, released on November 16, 2023, captures information pertinent to the COVID-19 epidemic. Our primary focus was leveraging this dataset for research during the COVID-19 outbreak, utilizing the 2015 data for comparative analysis. The 2015 data can be accessed through CHARLS (link: https://charls.charlsdata.com/pages/data/2015-charls-wave4/zh-cn.html). Similarly, the 2020 dataset is available through this link: https://charls.charlsdata.com/pages/data/2020-charls-wave5/zh-cn.html. This study received ethical approval from the Ethics Review Committee of Peking University (IRB 00001052-11014) and obtained written informed consent from all participants.

From the CHARLS database, we gathered various variables, encompassing age, gender, residential area (rural or urban), education level (ranging from no education/primary school to middle school and university or higher), marital status, smoke, drink, hypertension, diabetes, heart diseases, stroke, and sleeptime (<7hours, 7-9hours and >9hours) (10).

### 2.2 The definition of depression

This research employed the Center for Epidemiological Studies Depression Scale-10 (CESD-10) to evaluate depression. Within the CHARLS questionnaire, participants responded to 10 items regarding depressive symptoms or behaviors over the preceding week. The total scale score was derived by summing the responses to these 10 items, ranging from 0 to 30. A higher score indicated more pronounced depressive symptoms. Drawing from prior study (11), this research set a benchmark of 10 points to delineate depressive symptoms. Participants scoring above 10 were considered to have depression, while those scoring below 10 were not.

### 2.3 Statistical analysis

Categorical variables were depicted as proportions (%), while continuous variables were presented as mean ± standard deviation (SD). To discern differences among groups, we employed one-way ANOVA (for normal distribution) and the chi-square test (for categorical variables). Univariate and multivariate logistic regression models were utilized to ascertain the odds ratio (OR) and 95% confidence interval (95% CI) concerning the relationship between sleeptime and depression. Results encompassed unadjusted, minimally adjusted (age, gender), multiple adjusted (age, gender, marital status, education level, and residential area), and fully adjusted (all covariates) analyses. Subgroup analysis was executed using a stratified linear regression model, examining potential modifications and interactions via likelihood ratio testing. Furthermore, we compared sleeptime and the risk of depression in participants aged below 60 years versus those over 60 years old. Subgroup analysis based on age was performed utilizing a multivariate logistic regression model. Sensitivity analysis involved the exclusion of outliers, defined as sleeptime values exceeding the average range by 2 SD, while assessing subgroup interactions through likelihood ratio testing. Additionally, we employed restricted cubic spline analysis to explore the correlation between sleeptime and depression risk.

All statistical analyses were executed using the R 4.2.2 statistical software package (http://www.r-project.org, The R Foundation) and the free statistical software version 1.5 (12). A significance level of p<0.05 was considered statistically significant.

## 3 Results

### 3.1 Baseline characteristics of participants

The 2020 CHARLS dataset encompassed data from 19,331 consecutive participants and 10507 participants in 2015 CHARLS dataset, the flowchart for participant screening could be found in **Supplementary Figure 1**. **Table 1** delineated the baseline characteristics of participants, categorized by the presence or absence of depression in the database. In 2020, amid the COVID-19 outbreak, the database findings revealed an average participant age of 61.6 ± 10.1 years, with 9061 male participants, 6571 reporting depressive symptoms, and an average sleeptime of 6.7 ± 2.3 hours. Contrarily, in 2015, during a non-epidemic period, data indicated an average participant age of 58.9 ± 9.8 years, with 5322 males and 3242 individuals experiencing depression symptoms. The average sleeptime at that time was 7.0 ± 2.1 hours.

**Table 1:** Baseline characteristics of participants in two CHARLS database.

Characteristic	Total (2020, n = 19316)	Without depression (2020, n = 12745)	With depression (2020, n = 6571)	*P*-value in 2020	Total (2015, n = 10507)	Without depression (2015, n = 7265)	With depression (2015, n = 3242)	*P*-value in 2015
Age (years), Mean ± SD	61.6 ± 10.1	61.4 ± 10.4	61.9 ± 9.4	0.003	58.9 ± 9.8	58.7 ± 9.9	59.2 ± 9.7	0.019
Gender, n (%)				< 0.001				< 0.001
Female	10255 (53.1)	6063 (47.6)	4192 (63.8)		5185 (49.3)	3285 (45.2)	1900 (58.6)	
Male	9061 (46.9)	6682 (52.4)	2379 (36.2)		5322 (50.7)	3980 (54.8)	1342 (41.4)	
Residential area, n (%)				< 0.001				< 0.001
Rural	4922 (25.5)	3702 (29.0)	1220 (18.6)		6346 (60.4)	4149 (57.1)	2197 (67.8)	
Urban	14394 (74.5)	9043 (71.0)	5351 (81.4)		4161 (39.6)	3116 (42.9)	1045 (32.2)	
Education level, n (%)				< 0.001				< 0.001
Never attended school and primary school	12550 (65.0)	7637 (59.9)	4913 (74.8)		5974 (56.9)	3830 (52.7)	2144 (66.1)	
Middle school	6326 (32.8)	4732 (37.1)	1594 (24.3)		4275 (40.7)	3208 (44.2)	1067 (32.9)	
University annd above	440 (2.3)	376 (3.0)	64 (1.0)		258 (2.5)	227 (3.1)	31 (1.0)	
Marital status, n (%)				< 0.001				< 0.001
No	3121 (16.2)	1844 (14.5)	1277 (19.4)		1144 (10.9)	673 (9.3)	471 (14.5)	
Yes	16195 (83.8)	10901 (85.5)	5294 (80.6)		9363 (89.1)	6592 (90.7)	2771 (85.5)	
Smoke, n (%)				< 0.001				< 0.001
No	11254 (58.3)	6985 (54.8)	4269 (65.0)		5710 (54.3)	3803 (52.3)	1907 (58.8)	
Yes	8062 (41.7)	5760 (45.2)	2302 (35.0)		4797 (45.7)	3462 (47.7)	1335 (41.2)	
Drink, n (%)				< 0.001				< 0.001
No	12396 (64.2)	7723 (60.6)	4673 (71.1)		6537 (62.2)	4340 (59.7)	2197 (67.8)	
Yes	6920 (35.8)	5022 (39.4)	1898 (28.9)		3970 (37.8)	2925 (40.3)	1045 (32.2)	
Hypertension, n (%)				< 0.001				< 0.001
No	11601 (60.1)	7877 (61.8)	3724 (56.7)		8285 (78.9)	5823 (80.2)	2462 (75.9)	
Yes	7715 (39.9)	4868 (38.2)	2847 (43.3)		2222 (21.1)	1442 (19.8)	780 (24.1)	
Diabetes, n (%)				< 0.001				0.001
No	16465 (85.2)	11025 (86.5)	5440 (82.8)		9935 (94.6)	6904 (95.0)	3031 (93.5)	
Yes	2851 (14.8)	1720 (13.5)	1131 (17.2)		572 (5.4)	361 (5.0)	211 (6.5)	
Heart diseases, n (%)				< 0.001				< 0.001
No	15305 (79.2)	10525 (82.6)	4780 (72.7)		9349 (89.0)	6567 (90.4)	2782 (85.8)	
Yes	4011 (20.8)	2220 (17.4)	1791 (27.3)		1158 (11.0)	698 (9.6)	460 (14.2)	
Stroke, n (%)				< 0.001				< 0.001
No	17935 (92.9)	11982 (94.0)	5953 (90.6)		10313 (98.2)	7152 (98.4)	3161 (97.5)	
Yes	1381 (7.1)	763 (6.0)	618 (9.4)		194 (1.8)	113 (1.6)	81 (2.5)	
Sleeptime (hours), Mean ± SD	6.7 ± 2.3	7.0 ± 2.2	6.1 ± 2.3	< 0.001	7.0 ± 2.1	7.3 ± 2.0	6.4 ± 2.3	< 0.001

### 3.2 Relationship between sleeptime and depression

**Table 2** outlined the findings from the univariate analysis within the two CHARLS database, while **Table 3** displays the results from the multivariate logistic regression model analysis in two databases. In both databases, there was a close link between sleeptime and depression. In the fully adjusted model, participants who slept less than 7 hours exhibited a reduced risk of depression when compared to those who slept between 7-9 hours or longer (OR: 1.00 vs. 0.51 vs. 0.47, p<0.001 in the 2020 database; OR: 1.00 vs. 0.50 vs. 0.43, p<0.001 in the 2015 database). Even after excluding the influence of sleeptime exceeding 2 SD, this trend persisted in **Supplementary Table 1** (OR: 1.00 vs. 0.49 vs. 0.47, p<0.001 in the 2020 database; OR: 1.00 vs. 0.54 vs. 0.47, p<0.001 in the 2015 database).

**Table 2:** Association of covariates and depression risk in two CHARLS database.

Characteristic	OR_95CI% in 2020	*P*-value in 2020	OR_95CI% in 2015	*P*-value in 2015
Age (years), n (%)				
<60	1 (reference)		1 (reference)	
≥60	1.21 (1.14~1.29)	<0.001	1.16 (1.07~1.26)	0.001
Gender, n (%)				
Female	1 (reference)		1 (reference)	
Male	0.51 (0.48~0.55)	<0.001	0.58 (0.54~0.63)	<0.001
Residential area, n (%)				
Rural	1 (reference)		1 (reference)	
Urban	1.80 (1.67~1.93)	<0.001	0.63 (0.58~0.69)	<0.001
Education level, n (%)				
Never attended school and primary school	1 (reference)		1 (reference)	
Middle school	0.52 (0.49~0.56)	<0.001	0.59 (0.54~0.65)	<0.001
University and above	0.26 (0.20~0.35)	<0.001	0.24 (0.17~0.36)	<0.001
Marital status, n (%)				
No	1 (reference)		1 (reference)	
Yes	0.70 (0.65~0.76)	<0.001	0.6 (0.53~0.68)	<0.001
Smoke, n (%)				
No	1 (reference)		1 (reference)	
Yes	0.65 (0.61~0.70)	<0.001	0.77 (0.71~0.84)	<0.001
Drink, n (%)				
No	1 (reference)		1 (reference)	
Yes	0.62 (0.59~0.67)	<0.001	0.71 (0.65~0.77)	<0.001
Hypertension, n (%)				
No	1 (reference)		1 (reference)	
Yes	1.24 (1.16~1.31)	<0.001	1.28 (1.16~1.41)	<0.001
Diabetes, n (%)				
No	1 (reference)		1 (reference)	
Yes	1.33 (1.23~1.45)	<0.001	1.33 (1.12~1.59)	0.001
Heart diseases, n (%)				
No	1 (reference)		1 (reference)	
Yes	1.78 (1.65~1.91)	<0.001	1.56 (1.37~1.76)	<0.001
Stroke, n (%)				
No	1 (reference)		1 (reference)	
Yes	1.63 (1.46~1.82)	<0.001	1.62 (1.22~2.16)	0.001
Sleeptime, hours				
<7				
7-9	0.46 (0.43~0.49)	<0.001	0.47 (0.43~0.52)	<0.001
>9	0.47 (0.43~0.52)	<0.001	0.44 (0.39~0.49)	<0.001

**Table 3:** Weighted odds ratios (95% confidence intervals) of depression and different sleeptime in different models in two CHARLS database.

	Cases/participants	Non-adjusted Model	Adjusted model 1*	Adjusted model 2**	Adjusted model 3***
Sleeptime, hours (2020)					
<7	3987 (9312)	1(Ref)	1(Ref)	1(Ref)	1(Ref)
7-9	1724 (6709)	0.46 (0.43~0.49)	0.49 (0.45~0.52)	0.49 (0.46~0.53)	0.51 (0.47~0.54)
>9	860 (3295)	0.47 (0.43~0.52)	0.5 (0.46~0.55)	0.47 (0.43~0.51)	0.47 (0.43~0.52)
*P*-trend		<0.001	<0.001	<0.001	<0.001
Sleeptime, hours (2015)					
<7	1732 (4236)	1(Ref)	1(Ref)	1(Ref)	1(Ref)
7-9	994 (4043)	0.47 (0.43~0.52)	0.49 (0.45~0.54)	0.50 (0.45~0.55)	0.50 (0.45~0.55)
>9	516 (2228)	0.44 (0.39~0.49)	0.45 (0.40~0.51)	0.43 (0.38~0.49)	0.43 (0.39~0.49)
*P*-trend		<0.001	<0.001	<0.001	<0.001

*: Adjusted for age and gender.

**: Adjusted for age, gender, marital status, education level, and residential area.

***: Adjusted for age, gender, marital status, education level, residential area, smoke, drink, hypertension, diabetes, heart diseases, and stroke.

**Figure 1:**
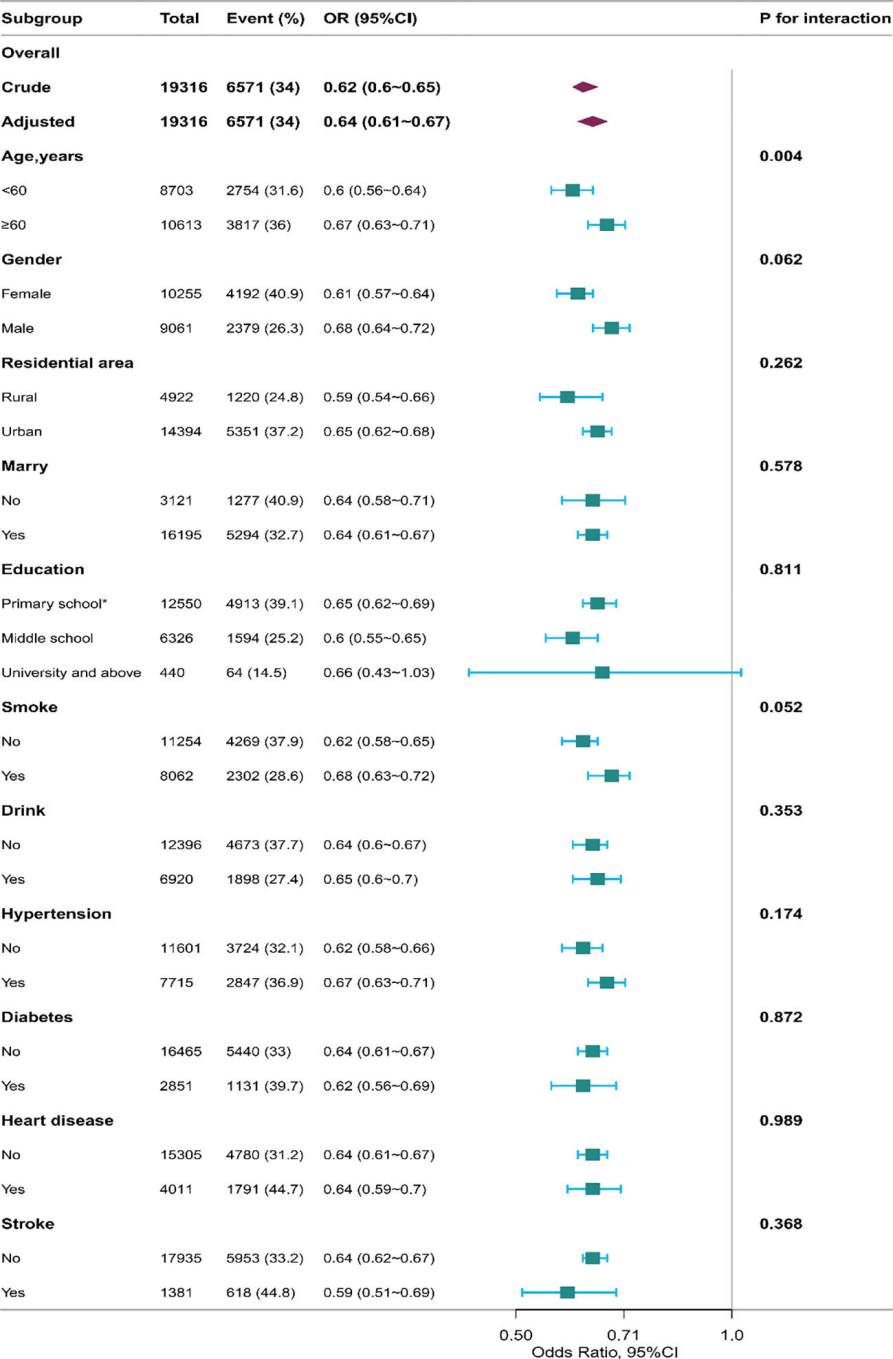
The relationship between sleeptime and the incidence of depression in the subgroup analysis in 2020 database. *: including never attended school and primary school. The results of subgroup analysis revealed a statistically significant interaction test for age (p for interaction = 0.004).

To bolster these findings, subgroup analysis was conducted, revealing a statistically significant interaction test for age (p=0.004, see **Figure 1**) in 2020 database. Hence, we conducted interaction analyses on both the unadjusted model (interaction p=0.033) and all adjusted variables (interaction p=0.004), revealing the relationship between age, depression, and various sleeptime, detailed in **Table 4**. Even after removing sleeptime more than 2 SD from the mean, this interaction persisted (unadjusted model: p=0.018 for interaction; adjusted all variables model: p=0.004 for interaction). For further specifics, **Supplementary Table 2** provides comprehensive information. Likewise, we examined the interplay between different sleeptime and depression within the 2015 database regarding age. The findings indicated a persistent interaction in both the unadjusted model (interaction p=0.046) and the adjusted all variables model (interaction p=0.026). After excluding sleeptime exceeding 2 SD, this interaction remained significant (unadjusted model, p=0.005 for interaction; adjusted all variables model, p=0.004 for interaction).

**Table 4:** Interactive effect of age in patients with depression in two CHARLS database.

Subgroups	Sleeptime, hours	Cases/participants	with depression		*P* for interaction	with depression*		*P* for interaction*
			Crude. OR_95CI %	Crude. P-value		Adjusted OR_95CI %	Adjusted *P*-value	
Age in 2020 (years)					0.033			0.004
<60 years	<7	1681 (4076)	1(Ref)			1(Ref)		
	7-9	769 (3351)	0.42 (0.38~0.47)	<0.001		0.45 (0.41~0.50)	<0.001	
	>9	304 (1285)	0.44 (0.38~0.51)	<0.001		0.45 (0.39~0.52)	<0.001	
*P*-trend		2754 (8703)	0.58 (0.54~0.62)	<0.001		0.60 (0.56~0.64)	<0.001	
≥60 years	<7	2306 (5245)	1(Ref)			1(Ref)		
	7-9	955 (3358)	0.51 (0.46~0.56)	<0.001		0.55 (0.50~0.61)	<0.001	
	>9	556 (200)	0.49 (0.44~0.55)	<0.001		0.49 (0.44~0.55)	<0.001	
*P*-trend		3817 (10613)	0.65 (0.62~0.69)	<0.001		0.67 (0.63~0.71)	<0.001	
Age in 2020 (years)					0.046			0.026
<60 years	<7	866 (2124)	1(Ref)			1(Ref)		
	7-9	540 (2328)	0.44 (0.39~0.50)	<0.001		0.46 (0.41~0.53)	<0.001	
	>9	233 (1125)	0.38 (0.32~0.45)	<0.001		0.38 (0.32~0.45)	<0.001	
*P*-trend		1639 (5577)	0.57 (0.53~0.62)	<0.001		0.58 (0.53~0.63)	<0.001	
≥60 years	<7	866 (2112)	1(Ref)			1(Ref)		
	7-9	454 (1715)	0.52 (0.45~0.59)	<0.001		0.55 (0.47~0.63)	<0.001	
	>9	283 (1103)	0.50 (0.42~0.58)	<0.001		0.50 (0.42~0.59)	<0.001	
*P*-trend		1603 (4930)	0.67 (0.62~0.73)	<0.001		0.68 (0.62~0.73)	<0.001	

*: Adjusted for age, gender, marital status, education level, residential area, smoke, drink, hypertension, diabetes, heart diseases, and stroke.

**Figure 2:**
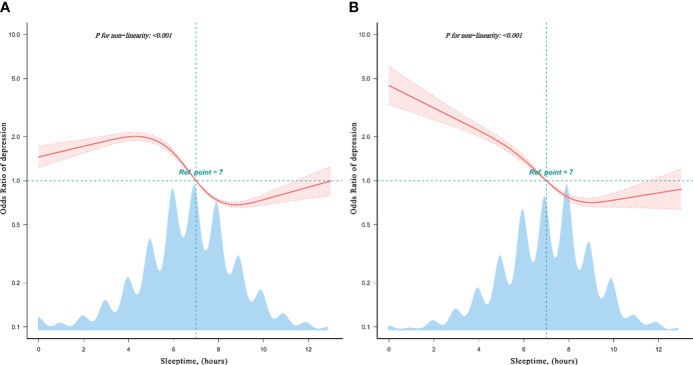
Restricted cubic spline model of the odds ratios of sleeptime with depression in CHARLS database. The restricted cubic spline analysis revealed both non-linear relationship in 2020 CHARLS database **(A)**, and in 2015 CHARLS database **(B)**, showcasing the different dose-response relationship between sleeptime and depression risk.

Additionally, the restricted cubic spline analysis across different databases of CHARLS in 2015 and 2020 (**Figure 2**) illustrated the non-linear relationship correlation and reveals varying temporal relationships between sleeptime and depression risk during non-COVID-19 and COVID-19 epidemics.

## 4 Discussion

The correlation between sleep time and depression has garnered significant attention within the academic community. Sleep, a complex phenomenon influenced by individual variances, living conditions, and other mental health factors, plays a pivotal role in the intricate interplay with depression. Previous research has consistently demonstrated a close connection between sleep quality, duration, and mental well-being, with insufficient or poor-quality sleep emerging as a potential risk factor for the onset and progression of depression (13). The findings of the present study reinforce and align with these established conclusions.

Against the backdrop of the COVID-19 pandemic, there has been a noticeable impact on people’s lifestyles and mental health. The investigation revealed a substantial disruption in sleep patterns during the pandemic. For individuals exposed to the COVID-19 pandemic, the virus could impact human pain perception and mental well-being via the immune response (14). Factors such as social isolation, heightened emotional stress, and information overload were identified as contributors to adverse sleep effects (9). This alteration in sleep patterns was found to be closely associated with an elevated risk of depression (8). While the link between sleep and depression is acknowledged, there remains some debate regarding the causative nature of this relationship. Some studies propose a unidirectional influence, suggesting that depression may lead to sleep disturbances, while simultaneously, sleep issues may exacerbate depressive symptoms (15). Consequently, this dynamic might be bidirectional, necessitating further research to elucidate the intricate causal dynamics between sleep and depression.

Insufficient sleep or poor sleep quality has been linked to depression (15). Moreover, extended periods of sleep have been associated with a heightened risk of depression (16). The data from this study amid the COVID-19 pandemic reveals an interaction among age groups (with 60 years as the cut-off), sleeptime, and depression. This implies potential differences in the correlation between sleep and depression among the elderly and younger populations. Among individuals under 60, the link between sleep and depression tends to be deemed more substantial, where inadequate sleep may amplify emotional distress (15). Studies indicate a notable correlation between sleep difficulties and depression incidence among young individuals (17). Emotional concerns may exert a stronger impact on the sleep quality of younger individuals, fostering a negative cycle.

As individuals age, sleep patterns may undergo alterations, leading to a more intricate relationship with depression. Some research indicates that shorter sleeptime in older adults correlates with an elevated risk of depression (18). Conversely, other studies suggest a connection between prolonged sleep and an increased risk of depression in older adults (16). Older individuals might exhibit greater susceptibility to changes in sleep quality over time. Sleep and depression might be linked to alterations in neurotransmitters. Serotonin, a neurotransmitter regulating emotions, cognition, and behavior, was closely tied to mental disorders like depression. As individuals age, serotonin synthesis and release might decline, particularly among the elderly. This reduction in serotonin levels might be associated with the onset and progression of emotional disorders like depression in older adults (19). Studies had indicated that dopamine system activity and norepinephrine levels diminish in the elderly, potentially correlating with cognitive decline, motor impairments, and emotional issues in later life (20, 21).

We perceive that the COVID-19 pandemic might have had a more pronounced impact on individuals’ mental well-being. Comparing the 2020 CHARLS data analysis findings to the 2015 dataset reveals a potentially stronger association between sleeptime and depression. This observation could be attributed to our focus on middle-aged and elderly populations in China. The unique circumstances of prolonged family isolation and pandemic management measures in China during the COVID-19 crisis provide a distinctive backdrop for our research.

Elderly individuals in China underwent a prolonged period of family isolation, an occurrence relatively uncommon during peacetime. These isolation measures, while lacking social interactions to some extent, also fostered a comparatively tranquil and stable family environment for the elderly. This setting might have positively influenced the psychological well-being of older adults. Amidst the pandemic, this environment likely encouraged introspection and contemplation, aiding in anxiety alleviation and managing negative emotions among older individuals (22). Furthermore, compared to social engagements, the family setting might offer more substantial support and a sense of security. During the epidemic, the prominence of mutual support and care within families likely contributed positively to the mental health of the elderly (23). The unity and nurturing atmosphere within families could potentially alleviate depression and offer enhanced psychological support. Further exploration of the relationship between the family environment and the mental well-being of the elderly during the COVID-19 crisis is warranted. A deeper comprehension of how family dynamics impact the mindset of older adults will aid in designing more effective prospective studies to enhance their mental health.

However, there were several limitations in this study that warrant discussion. Firstly, our study focused on data derived from the middle-aged and elderly population in China, potentially limiting the generalizability of the findings to other age cohorts and diverse populations in different geographical regions. Secondly, certain survey outcomes relied on self-reported data from the respondents, which can introduce recall bias. Thirdly, despite controlling for several covariates in this study, unaccounted factors may persist, necessitating further investigation in future research endeavors.

## 5 Conclusion

Whether during the COVID-19 pandemic or in non-pandemic times, individuals across various age groups might respond differently to different sleeptime, affecting their risk of depression. This highlights the importance of taking into account sleep patterns and age-related factors when devising prevention and intervention strategies. Doing so aims to foster a more comprehensive approach to promoting mental health on an individual level.

## Data availability statement

The datasets presented in this study can be found in online repositories. The names of the repository/repositories and accession number(s) can be found in the article/supplementary material. Further inquiries can be directed to the corresponding author/s.

## Ethics statement

The studies involving humans were approved by The Ethical Committees of Peking University. The studies were conducted in accordance with the local legislation and institutional requirements. The participants provided their written informed consent to participate in this study.

## Author contributions

CD: Writing – review & editing, Writing – original draft, Validation, Conceptualization. CW: Writing – review & editing, Writing – original draft, Formal analysis, Data curation. ZWL: Writing – review & editing, Writing – original draft, Formal analysis, Data curation. NB: Writing – review & editing, Writing – original draft, Formal analysis, Data curation. JZ: Writing – review & editing, Writing – original draft, Formal analysis, Data curation. AA: Writing – review & editing, Writing – original draft, Software, Methodology. YG: Writing – review & editing, Writing – original draft, Software, Methodology. XZ: Writing – review & editing, Writing – original draft, Software, Methodology. YY: Writing – review & editing, Writing – original draft, Software, Methodology. ZXL: Writing – review & editing, Writing – original draft, Validation, Conceptualization. CM: Writing – review & editing, Writing – original draft, Validation, Funding acquisition, Conceptualization.
