# Supplementary material for: The relationship between sleeptime and depression among middle-aged and elderly Chinese participant during COVID-19 epidemic and non-epidemic phases
Source: Front Psychiatry. 2024 May 10;15:1361184. doi: 10.3389/fpsyt.2024.1361184 (PMC11117139; doi:10.3389/fpsyt.2024.1361184)
Supplement: Supplementary file 1 [file DataSheet_1.pdf]

Supplemental table 1 Weighted odds ratios (95% confidence intervals) of depression and different sleeptime in different models in two database (excluded sleeptime ± 2SD) .

	Cases/participants	Non-adjusted Model	Adjusted model 1*	Adjusted model 2**	Adjusted model 3***
Sleeptime, hours (2020)					
< 7	3661 (8531)	1(Ref)	1(Ref)	1(Ref)	1(Ref)
7-9	1724 (6709)	0.46 (0.43~0.49)	0.48 (0.45~0.52)	0.48 (0.45~0.52)	0.49 (0.46~0.53)
> 9	765 (2931)	0.47 (0.43~0.52)	0.49 (0.45~0.54)	0.46 (0.42~0.51)	0.47 (0.42~0.51)
P-trend		<0.001	<0.001	<0.001	<0.001
Sleeptime, hours (2015)					
< 7	1431 (3722)	1(Ref)	1(Ref)	1(Ref)	1(Ref)
7-9	994 (4043)	0.52 (0.47~0.58)	0.54 (0.49~0.59)	0.54 (0.49~0.6)	0.54 (0.49~0.6)
> 9	466 (2026)	0.48 (0.42~0.54)	0.49 (0.43~0.55)	0.47 (0.41~0.53)	0.47 (0.41~0.53)
P-trend		<0.001	<0.001	<0.001	<0.001

*:Adjusted for age and gender.

**:Adjusted for age, gender, marital status, education level, and residential area.

***:Adjusted for age, gender, marital status, education level, residential area, smoke, drink, hypertension, diabetes, heart diseases, and stroke.

Supplemental table 2. Interactive effect of age in patients with depression in two dabase (excluded sleeptime mean ± 2SD).

Subgroups	Sleeptime, hours	Cases/participants	with depression Crude. OR_95CI %	Crude. P-value	P for interaction	with depression* Adjusted OR_95CI %	Adjusted P-value	*P* for interaction*
Age (years, in 2020)					0.018			0.004
	< 7	1611 (3867)	1(Ref)			1(Ref)		
< 60 years	7-9	769 (3351)	0.42 (0.38~0.46)	<0.001		0.44 (0.4~0.49)	<0.001	
	> 9	282 (1202)	0.43 (0.37~0.5)	<0.001		0.43 (0.37~0.5)	<0.001	
P-trend		2662 (8420)	0.57 (0.53~0.61)	<0.001		0.58 (0.54~0.62)	<0.001	
	< 7	2050 (4664)	1(Ref)			1(Ref)		
≥60 years	7-9	955 (3358)	0.51 (0.46~0.56)	<0.001		0.54 (0.49~0.6)	<0.001	
	> 9	483 (1729)	0.49 (0.44~0.56)	<0.001		0.5 (0.44~0.56)	<0.001	
P-trend		3488 (9751)	0.65 (0.62~0.69)	<0.001		0.67 (0.63~0.71)	<0.001	
Age (years, in 2015)					0.005			0.004
	< 7	761 (1941)	1(Ref)			1(Ref)		
< 60 years	7-9	540 (2328)	0.47 (0.41~0.53)	<0.001		0.49 (0.42~0.56)	<0.001	
	> 9	212 (1039)	0.40 (0.33~0.47)	<0.001		0.39 (0.32~0.47)	<0.001	
P-trend		1513 (5308)	0.59 (0.54~0.64)	<0.001		0.59 (0.54~0.64)	<0.001	
	< 7	670 (1781)	1(Ref)			1(Ref)		
≥60 years	7-9	454 (1715)	0.6 (0.52~0.69)	<0.001		0.61 (0.53~0.71)	<0.001	
	> 9	254 (987)	0.57 (0.48~0.68)	<0.001		0.56 (0.47~0.67)	<0.001	
P-trend		1378 (4483)	0.73 (0.67~0.79)	<0.001		0.73 (0.67~0.79)	<0.001	

*:Adjusted for age, gender, marital status, education level, residential area, smoke, drink, hypertension, diabetes, heart diseases, and stroke.

Supplemental figure 1 flow chart of participants in 2015 and 2020 CHARLS database.

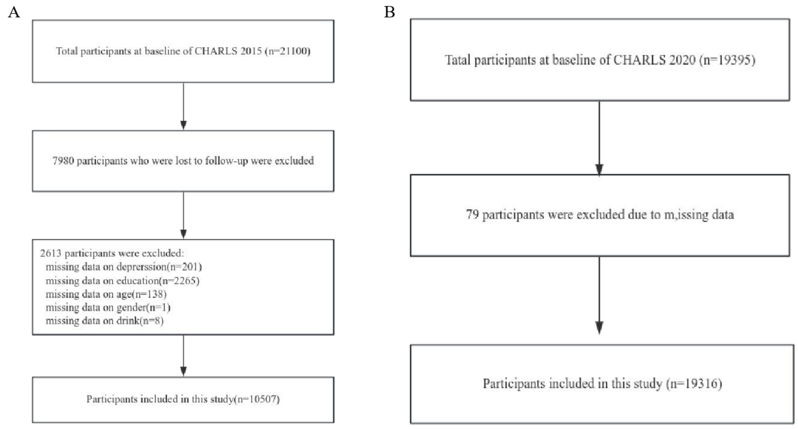

The 2015 CHARLS dataset encompassed data from 10507 consecutive participants(B) and 19,331 participants in 2015 CHARLS dataset(A).
